# Assessing the Food Safety Risk Posed by Birds Entering Leafy Greens Fields in the US Southwest

**DOI:** 10.3390/ijerph17238711

**Published:** 2020-11-24

**Authors:** Jorge M. Fonseca, Sadhana Ravishankar, Charles A. Sanchez, Eunhee Park, Kurt D. Nolte

**Affiliations:** 1 United States Department of Agriculture—Agricultural Research Service, Beltsville Agricultural Research Center, Food Quality Laboratory, Beltsville, MD 20705, USA; Eunhee.Park@usda.gov; 2 School of Animal & Comparative Biomedical Sciences, University of Arizona, 1117 E. Lowell Street, Tucson, AZ 85721, USA; Sadhravi@arizona.edu; 3 Maricopa Agricultural Center, Department of Environmental Sciences, The University of Arizona, 37860 W. Smith-Enke Rd, Maricopa, AZ 85138, USA; Sanchez@ag.arizona.edu; 4 Food and Drug administration, Center for Food Safety and Applied Nutrition, Produces Safety Network-Western Region, College Park, MD 20740, USA; Kurt.Nolte@fda.hhs.gov

**Keywords:** coliforms, *E. coli*, irrigation water, migratory birds, *Salmonella* sp.

## Abstract

In the US Southwest, it is common to observe birds in leafy green fields, though the risk they contribute to foodborne outbreaks remains unclear. In this study, we investigated and recorded the relationship between birds near leafy green fields and the risk for contaminated irrigation water or leafy green plants. We monitored the presence of birds for over two years and performed cloacal swab analysis for non-pathogenic *Escherichia coli*, *E. coli* O157:H7 and *Salmonella* *enterica*, while also monitoring the incidence of other microbial indicators. We also assessed the risks from bird feces by performing observations in a commercial field reported with *Salmonella* positive samples and by analyzing the survival of foodborne pathogens in bird feces. Our results showed that most of the birds near the crop fields were resident small birds. We did not observe a correlation between the number of birds in sites and the incidence of indicator bacteria (e.g., coliforms, *E. coli*) in irrigation canal water, with the exception of one out of four sites where water flow was low or stagnant. Using walk-in-traps, 305 birds were captured and placed in short-term captivity to determine the presence of various bacteria. None of the birds tested positive for *E. coli* O157:H7 or *Salmonella*. However, nearly 40% of the birds captured were confirmed positive for non-pathogenic *E. coli*. We found no correlation between age (young, adult, unknown), gender (male, female, unknown) and the incidence of *E. coli* positive birds, but we observed significantly higher probability of incidence during October–December. The role of relative humidity and temperature on bacterial survival appeared to play a key role in the survival of *Salmonella* on the leaves of spinach plants in a commercial field. This was also confirmed in laboratory conditions where *Salmonella* inoculated in bird feces and exposed to 15 °C and 80% RH(Relative humidity) survived beyond 133 days, while at 26 °C and 40% RH, the organism was undetectable after 63 days. Our results suggest that local birds associated with leafy green fields likely pose a minimal impact of risk for food contamination, but also points out the need for increased analysis specifically for *E. coli* O157:H7. Furthermore, our study suggests the need for expanding research that addresses risks associated with large migratory birds, especially in areas where stagnated water sources would be used for overhead sprinkle irrigation.

## 1. Introduction

Leafy greens consumed fresh and in ready-to-eat salads have been an important part of the post-second world war diet in the United States. They provide a significant source of nutrients and contribute to the recommended daily intake of 400 g of fresh produce [1]. The majority of the production of leafy green crops in the United States takes place in central California (primarily from April through October), and in Yuma, Arizona and southern California. The latter two locations constitute nearly three quarters of all of the nation’s leafy vegetable consumption from November to April [2,3].

While leafy greens are considered fundamentally nutritious, they also raise food safety concerns. In the United States, over nine million people suffer from a foodborne illness annually. More illnesses have been attributed to leafy greens than to any other food commodity with over 1/5 of all illnesses reported. The consumption of leafy greens was also the second most frequent cause of hospitalizations and the fifth most frequent cause of deaths between 1998 and 2008 [4]. In response, the US Food and Drug Administration (FDA) Food Safety Modernization Act (FSMA) established measures to monitor possible sources of contamination including animal intrusion and irrigation water quality [5]. Despite these measures, the FDA and the Centers for Disease Control and Prevention (CDC) reported that 40 foodborne outbreaks that occurred in 2009–2018 were linked to leafy greens, prompting the regulatory institutions to further call for immediate prevention and response actions. The report further revealed the immediate need to address important research gaps in the current understanding of contamination in the produce-supply chain [6].

Several of these outbreaks linking pathogenic bacteria (e.g., *Escherichia coli* O157:H7) with the consumption of leafy greens in central California have been traced back to mammals transiting from top mountain forest to low-level water sources [7], but could also be due to runoff, given the proximity of cattle production to crop fields, as has been indicated for other areas [8]. In Arizona and southern California, the presence of cattle is not prominent, rain events are scarce in the desert, and mammals seem not to pose the same degree of risk as compared to central California. However, the presence of birds in fields has been frequently observed [9], and there has not been any in-depth analysis that provides an insight on whether birds pose any contamination risk to leafy greens grown in the Southwestern USA. Studies with other animals (feral swine, cattle) help increase our overall understanding of the probable risk of avian-to-vegetable contamination [10,11], however, little is known about the actual foodborne risk originated from birds. 

Birds have been suspected to be carriers of foodborne pathogens in fields of vegetable crops that are eaten raw. Migratory birds are of particular concern as their potential to carry foodborne pathogens is well documented [12,13]. Migratory birds may carry antibiotic-resistant pathogens [14] in part due to a tendency of these birds to search food in close proximity to contaminated urban areas. However, domestic or garden birds also have been shown to be the source of human Salmonellosis [15]. An analysis of multiple studies with birds from different taxonomic orders [16] reported that birds from the Columbiform order have shown over 50% incidence of *E. coli*. However, the incidence of *Escherichia coli* O157:H7 in birds was found to be very low [17].

When analyzing the potential risk for birds to contaminate leafy greens there are several aspects that need to be taken into consideration. Shedding of pathogens could fluctuate across seasons. Cattle, for example, tend to shed *E. coli* O157:H7 for longer time and in higher incidence during the summer months, which appear to have an impact on the elevated incidence of outbreaks that have occurred during the consequent fall months [18,19]. In poultry, seasonality is also observed with higher prevalence of *Salmonella enterica* in the summer months [20]. Numerous studies have supported the association of wildlife, clinical pathogens, and antimicrobial resistance, in the food supply chain [14,21,22]. Wild birds have been identified as the sources of *Salmonella*, *E. coli* and *Campylobacter* [22,23], often in close proximity to leafy green production areas in the US west coast [24].

When an animal is constantly exposed to human enteric pathogens, it becomes important to determine to what extent the pathogens can be shed in the animal gut and for how long the pathogen survives in the feces excreted in an open field. In a critical scenario, animal feces can be deposited in irrigation water which can carry the pathogens and disperse the contamination in soil (through furrow irrigation or rain) or on leaves (through sprinkle irrigation). The survival of microbes in crops may be dependent on relative humidity, which is generally low in the Southwestern desert, and temperature, which is also lower than in central California during the growing season. However micro-climates in the plant canopy may be diverse due to plant density and irrigation frequency. The survival of foodborne pathogens in soil and leaves has been shown to last up to three weeks depending on environmental conditions and the irrigation system [25,26].

While it is important to recognize that the risk for carrying foodborne pathogens may vary among different types of birds, overall, birds may carry food safety risks for several reasons: (i) birds such as pigeons live in close association with cattle, which are known carriers of foodborne pathogenic bacteria; (ii) many bird species naturally migrate/roam from urban settings to agricultural fields; (iii) there is evidence of “spikes” in bacterial population in the water in irrigation canals at different times of the year, and the likely source of the bacteria is attributable to animal feces [25]; and (iv) bird feces are sometimes visually observed on the surfaces (soil, plants) of leafy green fields. Therefore, this research addressed the question whether birds roaming in or nearby crop fields can carry common foodborne pathogens. Furthermore, in the eventuality of pathogenic-bacteria shedding birds, we aimed at contributing to understand the potential for the survival of pathogens in feces or directly in plants.

The goal of our study was to determine the potential food safety risk posed by birds that are typically observed within leafy green vegetable fields in Yuma/Imperial counties in the Southwestern United States by estimating the potential prevalence of *E. coli* O157:H7 and *S. enterica* among these birds. The specific objectives were (i) to determine the risk in connection with birds’ characteristics (e.g., species, gender, age), (ii) assess the value of using indicator bacteria for mass monitoring the risk posed by birds, (iii) analyze the risk level carried by confirmed pathogen-positive fecal droppings in commercial conditions, and (iv) investigate the survival of foodborne pathogens in bird fecal material under various environmental conditions. 

## 2. Materials and Methods

This study was conducted in different locations in Yuma county, AZ and Imperial county, CA, as well as in the University of Arizona food safety laboratories located at the Agricultural Center in Yuma, AZ and at the main campus in Tucson, AZ. 

**Figure 1:**
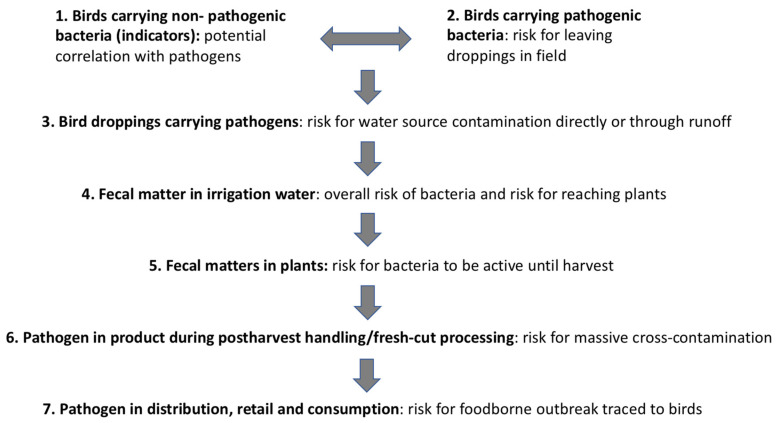
Presumable flow of transmission of a pathogen introduced by birds in the food chain and the eventual risk for an outbreak.

Our study looked at filling the information gap regarding the potential contamination by bird-shed pathogenic bacteria in the upstream of the food production-supply chain of leafy greens (points 1–5 in Figure 1). The scope of the study did not include further steps in the downstream side of the supply chain (postharvest handling, retail, consumption). 

### 2.1. Ethics Statement

Wildlife scientific collection permits were obtained from the United States Fish and Wildlife Service (USF&WS office in Phoenix, Arizona) to capture birds and retain them for 30 days to collect cloacal fecal samples. Permission was renewed yearly upon annual reports provided to the USF&WS. This study included the utilization of animals as study subjects, following the normative guidelines prescribed by the USF&WS and the approved procedures of the University of Arizona. We also obtained permission to access commercial fields from the fresh produce companies that owned the harvested crops. We sourced GPS(Global positioning system) data to locate plants previously spotted with *Salmonella* contamination from the third-party audit laboratory which had received clearance from the crop owners. 

### 2.2. Bird Sampling 

To capture birds in areas surrounded by crop fields we used rectangular wooden walk-in traps (approx. 90 × 60 × 30 cm) with dry food. The traps were placed in the Yuma area at four different locations, which represented different distances in regards to small animal operations, urban settings and irrigation canals, all three factors relevant when determining the potential cross-contamination risks. Site 1 was located next to a main primary canal that crosses the city of Yuma (32.7057971, −114.6930998). The closest leafy greens field was approximately 300 m away with residential areas in the vicinity, and a turbidity rate of the canal water of 2.4–2.9 NTU (Oakton). Site 2 was located in the limits of the city of Yuma near a small ranch with multiple domestic animals (e.g., poultry, horses, cattle), 100 m from a secondary canal and a crop field (32.7168403, −114.6892770). The turbidity rate of water in the irrigation canal was 3.0–3.7 NTU. Site 3 was in a commercial field located 8 km southwest from the city of Yuma and next to a (“lateral”) secondary irrigation canal (32.5814703, −114.7612035). The turbidity of the water in the irrigation canal ranged from 4.2–4.8 NTU. Site 4 was at the Yuma Agricultural Research Center—5 km east from the city of Yuma (32.7056295, −114.7019568), with secondary irrigation canals crossing research and commercial fields within 10 m. The turbidity of the water ranged from 3.2–4.0. The captured birds were released 48 h after fecal sampling unless the birds were found to be positive for either *Salmonella* or *E. coli*. A small mark (with biodegradable dye) was painted on the wings of the birds to prevent collecting the same bird more than once. Sampling was initially conducted with cloacal swabs using the BBL CultureSwab collection and transport system. The specimens were transported and processed for analysis of pathogens and indicator bacteria at the Yuma Agricultural Center within 4 h of collection. One swab was used to screen for *E. coli* O157:H7 and another to screen for *S. enterica*. The monitoring of the traps was conducted with the assistance of students from the Arizona Western College who had received training on handling and identifying birds. Continuous sampling was conducted for over two years. The sampling protocol was changed at the initial stage given the fact that the small mass obtained with the cloacal swabs was not sufficient to conduct analyses for both pathogens. Thus, the birds needed to be placed in cages for at least 1 day. The next day the most recently deposited feces (identified by moisture content appearance) were sampled. The first swab was streaked into test tubes containing 5 mL of Revive media (Neogen); the second swab was streaked 3 times into a MacConkey Agar plate (Millipore Sigma), and the third swab placed into a test tube containing 5 mL of Tryptic Soy Broth (Sigma-Aldrich).The birds that tested positive for *E. coli* were kept in individual cages for an additional 30 days to determine the *E. coli* recovery period from the feces of the birds. We recorded the bird species, gender and the approximate age (i.e., young or adult). The traps were kept in the site for approximately 3 h during the morning period. Given that the traps were set up at irregular intervals (not placed on a daily basis, but ranging from 2–4 times per week) and the number of days the traps were set up differed from one month to another (minimum of 8, and maximum of 16), the results were grouped based on bacterial analysis data from two months when less than 10 birds were captured in a single month. 

### 2.3. Monitoring the Incidence of Birds, Insects, and Bacterial Indicators in Selected Sites

In addition to capturing and releasing birds, we also monitored the number of birds in the same four sites where the traps were placed (approximately 10 m radius from traps) on 16 different dates. Insect traps (sticky pads of 12.7 × 17.8 cm at 50 cm above ground and attached to a wooden stick) were placed in the four sites for 24 h prior to the counting of the birds, which was conducted for one hour in the morning. Insects caught were counted to determine any relationship with the number of birds. Given that the traps had the limitation of capturing primarily small granivorous birds, counting the non-captured birds gave us an additional insight into what type of birds were present in the vicinity. In addition, water samples were taken from the nearby irrigation canal on the day birds were counted. Collection and laboratory analysis were performed following the procedure described by [25]. Water samples (100 mL) were taken in sterilized containers and submitted within 2 h to the food safety laboratory, where analysis of bacteria (non-pathogenic *E coli*, total coliforms, fecal coliforms,) was conducted following specifications of the Membrane Filtration methodology m-Coliblue 24 (USEPA method No. 10029; Hach, Loveland CO).

### 2.4. Analysis of Pathogenic Bacteria

For *Salmonella* identification, the test tubes containing Revive media were placed into a water bath kept at 35 °C and shaken at 135 rpm for 4 h. Then, the contents from the tubes were transferred into 5 mL of previously warmed Rappaport Vassiliadis (Thomas Scientific) broth and incubated overnight at 42 °C. The following day, the tube containing 5 mL of Revive + 5 mL of Rappaport Vassiliadis media, were cooled to room temperature. We followed the manufacturer’s recommendations for determination of the test results. For confirmation of these initial results the procedure was repeated, with samples simultaneously streaked on a CHROMagar *Salmonella* (CHROMagar Microbiology), which was incubated at 35 °C for 24 h in the dark. Presumptive positive samples were streaked onto Tryptic Soy Agar and incubated at 35 ± 20 °C for 24 h.

For confirmation of *Salmonella*, several tests were performed (detailed information in Bergey’s Manual): (i) The Urease test (inoculating sample into tubes of urea broth with an uninoculated control and incubating for 24 ± 2 h at 35 °C); (ii) lysine decarboxylase broth test. If lysine iron agar (LIA) was positive, then further testing was not required. A check for lysine decarboxylase was conducted by inoculating sample on lysine decarboxylase broth with a small amount of growth from triple sugar iron (TSI) slant. Incubation was in a tightly sealed plate at 35 °C for 48 ± 2 h. *Salmonella* positive samples were identified due to the alkaline reaction and the visible purple color. Negative results were identified as a yellow color. If neither color was detected, a few drops of 0.2% bromcresol purple dye were added and the reaction was re-monitored. Methyl Red-Voges-Proskauer (MR-VP) agar was used to plate each unclassified TSI (Triple Sugar Iron) slant suspected to contain *Salmonella,* then incubated for 48 ± 2 h at 35 °C. (iii) Other tests performed in early stages and with previously confirmed *Salmonella* were Voges-Proskauer (VP) and Methyl Red and Simmons citrate agar. The latter tests were discontinued based on the consistent results obtained with all tests (negative and positives).

For *E. coli* O157:H7 analysis, the feces samples (0.1 mL) suspended in sterile saline (0.15 NaCl) were spread plated directly onto Sorbitol MacConkey Agar (SMAC), and incubated at 35 °C overnight. Typical colonies from SMAC plates were transferred to tubes containing 3 mL of tryptic soy broth (TSB) and placed for incubation in a shaking water bath (135 rpm) overnight at 35 °C. The next day, samples were plated onto *E.coli*/Coliforms Petrifilm (3M) plates. Once samples were found presumptive positive, other colonies from the SMAC plates were grown in TSB to make a glycerol stock and were kept stored at –80 °C until the procedure for confirmation of *E. coli* O157:H7 was performed. For confirmation, three tests were performed simultaneously (as a conclusion to maximize accuracy following an analysis conducted with cow feces that contained the pathogen): (1) Presumptive positive colonies from SMAC were tested for the IMViC (Indole methyl red Voges-proskauer citrate) reactions (Kovac’s reagent) and inoculated into tubes with Lauryl Sulfate Tryptose (LST) broth to confirm gas production; (2) Voges-Proskauer (VP) reactive compounds. Inoculations of tubes containing MR-VP broth and incubation for 48 ± 2 h at 35 °C were followed by transferring 1 mL to 13 mm × 100 mm tubes, with an addition of 0.6 mL-naphthol solution and 0.2 mL 40% KOH, and mixing. A few crystals of creatine were added, shaken and allowed to incubate for 2 h. The test was considered positive if eosin (pink) color developed. After the VP test, MR-VP tubes were incubated additionally for 48 ± 2h at 35 °C, followed by an addition of 5 drops of methyl red solution to each tube. Distinct red color showed positive results for the test. Yellow was considered a negative reaction; (3) citrate and lactose. Tubes containing Koser’s citrate broth were inoculated and incubated for 96 h at 35 °C. Development of distinct turbidity was considered a positive reaction. Inoculation of tubes containing LST and incubation for 48 ± 2 h at 35 °C was done. As with Kovac’s reagent tests, in this case we confirmed the presence of bacteria by identification of gas production.

### 2.5. Field Sampling in Commercial Field

During the process of this research, a commercial field was left unharvested in Holtville, Imperial county, California (50 km distance from Yuma; exact location not disclosed as per agreement with the grower and owner of the harvested field) as the third-party auditing laboratory reported numerous *Salmonella* positive samples. The location of the crop field is known for being part of a major corridor of migratory birds, which normally stay in the area for several days/weeks during winter months. The field (an approximate area of 4 hectares) was sampled (20 samples) following the exact GPS coordinates provided by the auditing laboratory. Coordinates were traced with a 10 cm accuracy using a Juno ST navigation device (Trimble). We noticed a higher agglomeration of plants and associated wetness in the center of the beds in comparison to those on the edges of the bed. Thus, the samples from the three sections (two edges and center) were considered separately for reporting results. Samples were taken from the field three and eight days after the submission of the report by the third-party auditing laboratory, which had taken plant samples 2–3 days earlier. 

### 2.6. Survival of Foodborne Pathogens in Bird Feces

The bacteria used for inoculation of feces were *E. coli* O157:H7 and *S. enterica*. Three strains of *E. coli* O157:H7 (F4546, 960218 and SEA 13B88) were used and made into a cocktail. *S. enterica* serotype Newport was used. Each bacterial culture was prepared by inoculating cryo-preserved cells in tryptic soy broth (TSB; Difco Becton Dickinson) and incubating overnight (18–20 h) at 37 °C with shaking at 150 rpm. Two transfers were done before a working culture was prepared. Cells were harvested by centrifugation (2000× *g* for 10 min) and washed twice in sterile buffered peptone water (BPW; Difco Becton Dickinson). Cells were finally suspended in BPW to a concentration of about 9 log CFU/mL and formed the initial inoculum needed for experiments. To prepare the cocktail for *E. coli* O157:H7, equal volumes (3 mL) of the inoculum suspended in BPW, of each strain, were dispensed in a test tube and mixed well.

Bird feces samples (100 g) were placed on a sheet of aluminum foil and pressed into a thin layer. Overnight culture of *Salmonella* Newport or *E. coli* O157:H7 cocktail (1 mL) was inoculated on the entire layer evenly. Samples were then dispensed into stomacher bags and mixed well by manually shaking for 1 min. Afterwards, the samples were stored at environmental conditions simulating the growing regions combinations of either 26 °C and 40% relative humidity or 15 °C and 80% relative humidity. Samples were taken at appropriate time intervals to enumerate the surviving bacteria. Feces samples (3 g) were transferred into centrifuge tubes and 27 mL of BPW was added. The mixtures were vortexed well for 5 min and serial dilutions were done as needed in BPW. Samples were plated on xylose lysine desoxycholate (XLD; Difco Becton Dickinson) agar with chloramphenicol (15 μg/mL) and tetracycline (10 μg/mL) for enumeration of *Salmonella* Newport, and on CHROMagar^TM^ O157 (Difco Becton Dickinson) for *E. coli* O157:H7. Plates were incubated at 37 °C for 24–48 h and counted. Three replicates were done.

### 2.7. Statistical Analysis

A correlation matrix for all measured parameters, including quality attributes and indigenous microorganisms was constructed using the CORR procedure of SAS (version 9.4, Cary, NC), which provided a value for the Pearson’s correlation (R) and the associated P value. In addition, Chi-square test of independence was conducted using the FREQ procedure of SAS to determine whether the incidence of *E. coli* are related to the various independent variables. The dependent variables (binary: yes/no) were incidence of birds with *E. coli,* and the predictor variables (as independent variable) included the month, the gender (female, male, and unknown), and the age (adult, young, and unknown). By grouping the months of the year (data taken in December, January and February were grouped as winter; that from March, April and May as spring; the June, July, August data as summer; and that from September, October, November as fall) a new variable namely ‘season’ was created for further analyses. The species of birds was not included due to insufficient amount of data (less than five) with some of the species. Data consisted of a total of 305 observations. The plotting of statistical results was created using SigmaPlot (version 13.0, Systat Software). Differences in the survival of the pathogens (*Salmonella* and *E. coli* O157:H7) between two temperature/relative humidity conditions were determined using a paired-samples t-test. The tests were performed using Microsoft Excel 2016 (Microsoft, Redmond, WA, USA) with a 0.05 level of significance.

## 3. Results

In this study, ten species of birds (Table 1) were captured with the walk-in traps located in four sites in the Yuma area. Most of these birds are known for sedentary behavior or are birds that have adopted the local area as their permanent habitat. Only three species were found with predominant seasonal migratory dynamics, including the great-tailed grackle (GTGR), the brown-headed cowbird (BHCO), and the yellow head blackbird (YHBL).

**Figure 2:**
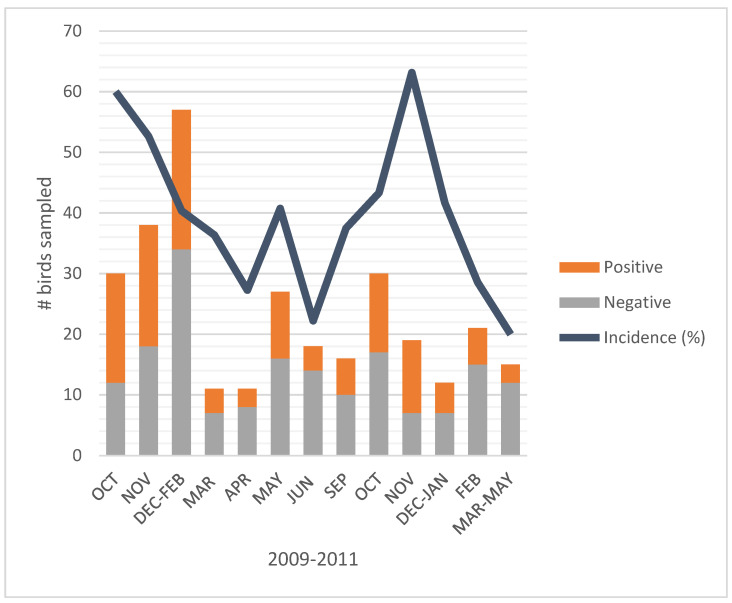
Incidence of birds found positive for generic non-pathogenic *Escherichia coli.*

Our study involved more than 300 birds captured through the walk-in traps, yet not a single bird showed positive results for *Salmonella* sp. (data not shown). Likewise, no bird was found shedding *E. coli* O157:H7 (data not shown). However, our laboratory analyses also showed that approximately 40% of the birds (Figure 2) carried non-pathogenic *E. coli*. Moreover, when we further analyzed the *E. coli* isolates, we did not confirm the presence of *E. coli* O157:H7 in any of the birds that had presented *E. coli* positive results.

**Figure 3:**
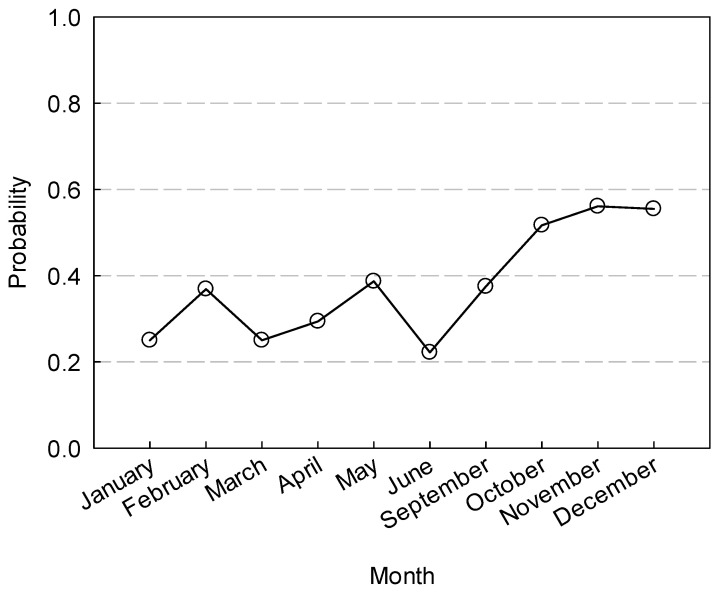
Monthly predicted probabilities (odd ratio) of the occurrence of non-pathogenic *E. coli* in birds roaming lettuce fields in the Yuma area. Actual individual observations were either “0.0” indicating no incidence of bacteria or “1.0” indicating bacteria was recovered.

The months of October, November and December showed the highest incidence of birds with non-pathogenic *E. coli*. The probability (odd ratio) of finding birds shedding *E. coli* during the last three months of the year was found to be at least 50% higher than that in previous months of the year (Figure 3). 

The statistical significance of this finding was further confirmed through a Chi-Square test of independence (Table 2).

**Figure 4:**
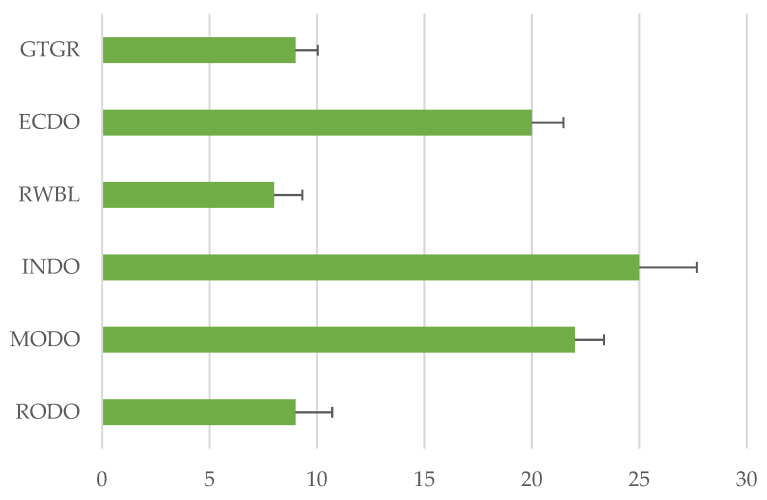
Average number of days birds in captivity remained shedding generic non-pathogenic *E. coli*. Error bars are the standard errors of the mean. Note: Birds were released on day 30, when MODO (22 out of 67), and INDO (8 out of 15) were still shedding *E. coli*. Number of birds in captivity for other species was 8 for GTGR, 24 for ECDO, 27 for RWBL and 15 for RODO.

There is a statistically significant association (dependent) between the incidence of birds with *E. coli* and season (*p* = 0.0112 at α = 0.05). MODO birds were distinctively high in captures and in shedding *E. coli.* Inca dove (INDO) was also associated with a long *E. coli* shedding period. In fact, in these two species the calculation of an average number of days that *E. coli* were shed per bird was not possible because many of the birds were still shedding *E. coli* at day 30 when the birds were released. Rock dove (RODO) and the red-winged black bird (RWBL) showed a rapid decline in *E. coli* shedding during captivity (Figure 4).

The bird counting survey confirmed that the great majority of birds associated with leafy greens fields in the Yuma area were small birds, all from the same species captured with traps. The assessed relationship (Pearson) between the number of birds and bacterial indicators, i.e., *E. coli* and fecal coliforms, in water was only significantly high (*p* < 0.03580 and 0.02720 respectively; *p* < 0.05) in one (Site 3) of the four locations monitored in our study (Table 3). In two sites (Sites 1 and 3) a correlation was also found between insect counts and bird counts. 

While the results obtained from monitoring birds in captivity or water samples in relation to birds indicated minimum food safety risk, the high numbers of larger migratory birds reported in a specific site in the Imperial county were associated with a cross-contamination of *Salmonella* in a field that was subsequently recalled and left unharvested. The evaluation of the field shortly after the third-party audit laboratory report showed a high incidence of *Salmonella* (40–87%). A second analysis conducted 8 days after the third-party audit report still recovered positive samples, and the recovery of *Salmonella* was near 50% in samples taken from the center section of the cropping beds, while samples from the edge of the beds were low (often non-detected) (Table 4).

**Figure 5:**
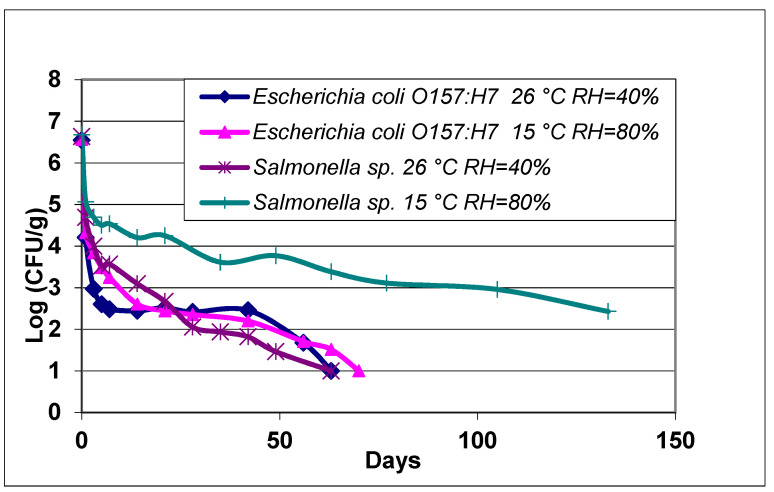
Survival of *Escherichia coli* O157:H7 and *Salmonella* Newport in feces collected from birds captured in Yuma fields.

The possible role of relative humidity within the crop canopy on *Salmonella* detection was evaluated in the laboratory and the results confirmed significantly longer period of recovery at a RH of 80% than at 40%. This was observed despite the fact that the latter RH was simultaneously at a closer to optimal temperature for bacterial growth (26 °C) than the former (15 °C). The recovery of *E. coli* O157:H7 was 7 days longer at 80% RH/15 °C than at 40% RH/26 °C. The analysis of *Salmonella* showed more pronounced differences, with samples at 80% RH/15 °C still recovering the pathogen after 132 days (presence at above 2 Log CFU/g), when there was no recovery of the bacteria at 40% RH/26 °C (Figure 5). The survival of *E. coli* O157:H7 at 26 °C/40% RH and 15 °C/80% RH was not significantly different (*p* > 0.05). A significant difference (*p* < 0.05) was observed for the survival of *Salmonella* at 26 °C/40% RH and 15 °C/80% RH.

## 4. Discussion

Our study shows that no small bird carried foodborne pathogens, which can be seen as a positive finding by all parties in the leafy greens supply-consumption chain. Despite the limitations of the trap system, we confirmed that there is a low probability that common birds roaming the Yuma leafy greens fields will carry *E. coli* O157:H7. This observation agrees with a report on local conditions [9] that showed that small birds in the proximity to livestock facilities did not carry *E. coli* O157:H7. One concern emerges in relation to the large number of birds that were found positive for generic *E. coli*. Despite the fact that these samples were confirmed to be non-pathogenic *E. coli*, continuous droppings in irrigation water can spike levels of generic *E. coli* and create unnecessary alarming signals to growers in the area, as they normally test for pathogen indicators (e.g., generic *E. coli*, total coliforms) and direct pathogen testing is not routinely performed. The impact of small birds carrying generic *E. coli* on the levels of bacteria in the nearby irrigation canal waters was not confirmed broadly in this study. Thus, our results are inconclusive in this aspect as no biomarker was used to track *E. coli* in water and correlate with *E. coli* harbored by birds. However, it is relevant to note that a spike in birds shedding generic *E. coli* was observed in the last three months of the year, not coinciding with the spike of *E. coli* in a comprehensive three-year water survey conducted in the Yuma area in the July–September months [26]. This could suggest that the spike in avian *E. coli* does not significantly impact the overall *E. coli* levels in irrigation canal waters in the Yuma area. Moreover, our results comparing the incidence of *E. coli* in birds and water samples positive for *E. coli* could be explained by the flow rate of nearby irrigation water. In the only location (site 3) where birds and bacterial indicators (fecal coliform, *E. coli*) showed a correlation, the water flow rate in the canal was nearly stagnant during all the sampling dates. The impact of water stagnation on water microbial quality has been demonstrated in different water systems including the piping for buildings’ tap water [27]. Flushing for certain time has been the recommendation to avoid the accumulated bacteria in the stagnant water. There are several reasons why lateral/secondary irrigation canals differ in water flow rates. Likely, the most important reason for stagnant waters in the US Southwest is the lack of synchronization of the irrigation schedules with that of main water supply out of the Colorado river, which results in water in the secondary canals not being utilized for days/weeks. Changes in weather, and subsequent fluctuation in plants’ water requirement prompt growers to have available water outside a fixed water distribution schedule from main canals.

In the same, Site 3, the observed relationship between birds and insects, which can be a source of cross-contamination [28,29] could also be attributed to water stagnation, as many insects lay their eggs in these types of waters.

The extensive cross-contamination of *Salmonella* sp. observed in the commercial baby spinach field may be seen in two ways. First, it supports the possibility that large migratory birds can act as a reservoir of the pathogen (we did not confirm it as DNA pathogen strain analysis of waterfowls and the bacteria in plants were not conducted). Coincidentally, the owner of the field had reported an unusual concentration of Canadian geese (*Branta canadensis*) in one section of the farm, in proximity to the irrigation canal. No other animals known for harboring *Salmonella* (e.g., reptiles) were reported. Secondly, this incident raises the question: in what way a relatively small flock of geese can spread contamination so massively across a large leafy green field? The likely explanation for the spread of contamination may be found again in connection with irrigation water that is precisely in stagnant conditions for one or several days before water is used for overhead sprinkle irrigation. Stagnation allows for the stability of sediments, which have been shown to be key for the prevalence of *Salmonella* in rivers, ponds and irrigation canals [30,31]. The capability of *Salmonella* to produce biofilm in aquatic environment may further explain the persistence of the pathogen in conditions similar to what is found in the irrigation canals in the area of this study [32,33]. Furthermore, *Salmonella* is known for its ability to develop desiccation resistance that can enhance survival under dry conditions in the soil or other dry environmental conditions [34]. The way the irrigation canal water could have been contaminated is uncertain, as it may have been a result of direct droppings of feces, through splashing of bird feces onto the soil, or runoff (there had been some rain in previous days) or all of the above. 

Large waterfowls from Canada and northern US migrate to the US Southwest coast during winter under diverse changing environmental conditions, which may expose them to higher risk for acquiring *Salmonella*, *Campylobacter* and antimicrobial resistant *E. coli* [22]. During this study we were informed by the USDA-Fish and Wildlife Services in Phoenix, AZ that an early storm (in October) froze lakes in southern Canada, Montana and surrounding areas which appeared to have caused an earlier migration of birds, particularly large birds such as geese and ducks. This also coincided with the unusual higher temperatures in the Sonoran desert during the fall season of the study. The early migration of waterfowl may result in birds searching for water and food in different scenarios, which may pose different risks including supplemental feeding in areas more exposed to pathogens including livestock and urban areas [35,36]. Additionally, when birds carry pathogens and defecate in warmer waters, the chances for the survival of pathogens in the water increases [25].

The reason for the rapid decline in bacterial shedding by some of the bird species, and by certain individuals within species, is uncertain, though it is likely due to variation among birds in responding to stress as hormonal signaling and stress-related compounds in the host organism interact with quorum sensing of bacteria which activates bacterial defense mechanism, including biofilm formation [37].

Our results reveal the different decline patterns of *Salmonella* according to the location of the plant material within the cropping bed. High survival of pathogenic bacteria has been observed in leafy green products in some studies. [38] observed that *E. coli* O157:H7 survived for over two weeks in protected tissue of lettuce under controlled production conditions. [39] observed no change in population of *E. coli* O157:H7 during refrigerated storage after inoculation was performed during postharvest handling, regardless of the type of lettuce and the irrigation system used in the field. These results highlight the potential impact of sunlight (ultraviolet radiation) and relative humidity (and the subsequent desiccation effect) on the population decline of bacterial pathogens. Low humidity in the edges of the cropping bed could be an important factor influencing the survival of the pathogens, which confirms the importance of prevention measures to reduce excess water in the field during the days before harvest. In this study, the effect of relative humidity was further confirmed in laboratory conditions. While the overall relative humidity in the US Southwest is generally low (on average commonly at or below 40% relative humidity), irrigation water and high density of plants could create a micro-climate that can increase the relative humidity. The significant increase in survival of *Salmonella* under these conditions call for a holistic approach that includes not only the monitoring of Canadian geese or other waterfowls along their migration routes [5], but also regulation of excess moisture during the days before harvest. If the termination irrigation (last irrigation prior to harvest) is appropriately scheduled, the weight loss of the eventual harvested product could be minimal, while it can simultaneously produce a positive effect on the sensory quality and postharvest shelf-life of the produce [40]. Precisely, timing of the last irrigation (in particular overhead sprinkle) and creation of buffer zones when animal feces are encountered were the key conclusions of a recent study in another lettuce production area [41]. For the US Southwest we provided evidence to better inform future regulatory standards. Our results add towards taking a holistic approach for handling food safety risk in connection with birds. This approach must include efforts to avoid waterfowls settling in critical areas such as irrigation water sources, notably when sedimentation and water stagnation is prevalent. Our study encourages grower’s responsiveness to birds roaming over fields based on the type of birds and the density of the avian agglomeration. Furthermore, when flocks of birds are inevitable in crop field areas, based on our findings, the analysis of irrigation water for specific foodborne pathogens (e.g., *Salmonella*) will be relevant, in addition to common bacterial indicators.

## 5. Conclusions

In this study, we assessed the risk posed by birds that enter leafy greens crop fields, and subsequently shed fecal droppings on plants, soil and water sources. We concluded that it is improbable that the non-migratory birds observed in our study, which prevail in the areas adjacent to the leafy greens fields in the US southwest, carry common foodborne pathogens (i.e., *E.coli* O157:H7, *Salmonella*). The high incidence of non-pathogenic *E. coli* observed (approximately 40%) confirms the importance of not relying solely on generic *E. coli* analysis to determine the risk of contamination. The analyses of a commercial field impacted by droppings of larger (migratory) birds confirmed the presence of *Salmonella*. The risk of migratory birds, however, needs further research, because it is highly unlikely that bird droppings shed directly on plants could result in extensive cross-contamination across the field. We have provided a discussion that elucidates the need for further research to understand potential ways in which the contamination spreads and investigate the inactivation dynamics of the pathogen under commercial field conditions. Our results inform future regulatory frameworks for better managing leafy green fields in relation to wild avian species habitats.

## Figures and Tables

**Table 1:** Species of birds captured in four sites of the Yuma area during the time period of the vresearch.

			Locations Where Birds were Found **				Total
Common Name	Alpha Codes *	Scientific Name (Order)	Site 1	Site 2	Site 3	Site 4	
**Rock dove**	RODO	*Columba livia* (Columbiformes)	13	0	1	4	18
**Mourning dove**	MODO	*Zenaida macroura* (Columbiformes)	49	27	19	84	179
**Inca dove**	INDO	*Scardafella inca* (Columbiformes)	16	2	2	19	39
**Red-winged black bird**	RWBL	*Agelaius phoeniceus* (Passeriformes)	2	5	1	19	27
**Great-tailed grackle**	GTGR	*Quiscalus mexicanus* (Passeriformes)	1	5	0	2	8
**House Sparrow**	HOSP	*Passer domesticus* (Passeriformes)	1	0	0	0	1
**Eurasian collared-dove**	ECDO	*Streptopelia decaocto* (Columbiformes)	6	4	6	8	24
**Common-ground dove**	COGD	*Columbina passerina* (Columbiformes)	4	0	0	0	4
**Brown-headed cowbird**	BHCO	*Molthrus ater* (Passeriformes)	3	0	0	1	4
**Yellow head blackbird**	YHBL	*Xanthocephalus xanthocephalus* (Passeriformes)	0	1	0	0	1
**Total**			95	44	29	137	305

* In accordance with the 60th American Ornithological Society Supplement (2019); ** site locations are described in methodology.

**Table 2:** Values of Chi-Square test of independence between positive samples for non-pathogenic *E. coli* and the site, season (summer, fall, winter, spring); gender (male, female, unknown); age (adult, young, unknown).

Variables	Chi-Square Value	*p*-Value
Site	7.923	0.244
Month	15.530	0.077
Season	11.098	0.011 *
Gender	0.608	0.738
Age	3.326	0.190

Significant result is indicated (* *p* < 0.05 at α = 0.05).

**Table 3:** Incidence of birds in four selected sites in the Yuma area, in relation to incidence of insects and bacterial indicators in water of nearby irrigation canals.

Variable	Bacteria Indicator	Four Selected Sites In Yuma			
		Site 1 (n = 16)	Site 2 (n = 16)	Site 3 (n = 16)	Site 4 (n = 16)
Bird	Total coliform	0.18 ^a^ (0.501) ^b^	−0.18 (0.493)	−0.09 (0.739)	0.04 (0.895)
	Fecal coliform	0.18 (0.505)	−0.14 (0.603)	0.59 (0.016) *	0.20 (0.463)
	*E. coli*	−0.02 (0.949)	−0.09 (0.731)	0.63 (0.008) **	0.03 (0.920)
Insect	Total coliform	0.08 (0.775)	−0.20 (0.466)	−0.01 (0.963)	0.10 (0.710)
	Fecal coliform	−0.16 (0.564)	0.30 (0.266)	0.26 (0.334)	0.01 (0.97)
	*E. coli*	−0.12 (0.650)	−0.08 (0.761)	0.53 (0.034) *	−0.06 (0.823)
Bird vs Insect		0.65 (0.007)**	−0.12 (0.671)	0.53 (0.038) *	−0.30 (0.258)

The significance levels of the Pearson correlation coefficients are indicated by * (*p* ≤ α = 0.05) or ** (*p* ≤ α = 0.01); ^a^ = correlation coefficient and ^b^ = *p*-value.

**Table 4:** Recovery of positive (P) samples (for *Salmonella*) in a commercial baby spinach field (Holtville, CA) after a third-party laboratory (TPL) assessment found widespread contamination in the field. Columns reflect location of the samples along the width (84 inches/214 cm) of the beds; A indicates 26″/66 cm on east edge; B: central 32″/82 cm; C: 26″/66 cm on west edge).

*Location in Bed ≥*	*A*	*B*	*C*	*A*	*B*	*C*
*Sample*	3 Days after TPL Report			8 Days after TPL Report		
1			P			N
2			N			N
3	P			N		
4	P			P		
5		P			P	
6		P			N	
7		P			N	
8			P			N
9			N			N
10		P			P	
11	N			N		
12	P			P		
13	N			N		
14	N			N		
15		P			N	
16		P			P	
17			N			N
18		P			P	
19		N			N	
20	N			N		
**Incidence of positive results**	**0.43**	**0.87**	**0.4**	**0.29**	**0.5**	**0**

## References

[1] Food and Agriculture Organization of the United Nations (FAO), World Health Organization (WHO) Fruit and vegetables for health: report of the Joint FAO/WHO Workshop on Fruit and Vegetables for Health, 1–3 September 2004, Kobe, Japan.

[2] Astill G. (2019). Seasonality in Romaine outbreaks and regional shipments. Situation and Outlook Report.

[3] Agricultural Marketing Service (2020). Specialty Crops Market News (Data sets). Custom Reports.

[4] Painter J.A., Hoekstra R.M., Ayers T., Tauxe R.V., Braden C.R., Angulo F.J., Griffin P.M. (2013). Attribution of foodborne illnesses, hospitalizations and deaths to food commodities by using outbreak data, United Sates, 1998–2008. Emerg. Infect. Dis..

[5] U.S. Food and Drug Administration (FDA) (2020). Standards for the Growing, Harvesting, Packing, and Holding of Produce for Human Consumption: Guidance for Industry.

[6] US Food and Drug Administration 2020 Leafy Greens STEC Action Plan. https://www.fda.gov/food/foodborne-pathogens/2020-leafy-greens-stec-action-plan.

[7] Jay M.T., Cooley M., Carychao D., Wiscomb G.W., Sweitzer R.A., Crawford-Miksza L., Farrar J.A., Lau D.K., O’Connell J., Millington A. (2007). Escherichia coli O157:H7 in feral swine near spinach fields and cattle, central California coast. Emerg. Infect. Dis..

[8] Pandey P.K., Kass P.H., Soupir M.L., Biswas S., Singh V.P. (2014). Contamination of water resources by pathogenic bacteria. AMB Express.

[9] Rivadeneira P., Hilson C., Justice-Allen A., Jay-Russell M. Pathogen Risks Related to the Movement of Birds Frequenting Livestock and Fresh Produce Growing Areas in the Southwestern U.S. 2016. Proceedings of the Vertebrate Pest Conference.

[10] Mishra A., Pang H., Buchanan R.L., Schaffner D.W., Pradhan A.K. (2016). A System Model for Understanding the Role of Animal Feces as a Route of Contamination of Leafy Greens before Harvest. Appl. Environ. Microbiol..

[11] Herman K.M., Hall A.J., Gould L.H. (2015). Outbreaks attributed to fresh leafy vegetables, United States, 1973–2012. Epidemiol. Infect..

[12] Foti M., Rinaldo D., Guercio A., Giacopello C., Aleo A., de Leo F., Fisichella V., Mammina C. (2011). Pathogenic microorganisms carried by migratory birds passing through the territory of the island of Ustica, Sicily (Italy). Avian Pathol..

[13] Benskin C.M.H., Wilson K., Jones K., Hartley I.R. (2009). Bacterial pathogens in wild birds: A review of the frequency and effects of infection. Biol. Rev..

[14] Cao J., Hu Y., Liu F., Wang Y., Bi Y., Lv N., Li J., Zhu B., Gao G.F. (2020). Metagenomic analysis reveals the microbiome and resistome in migratory birds. Microbiome.

[15] Lawson B., de Pinna E., Horton R.A., Macgregor S.K., John S.K., Chantrey J., Duff J.P., Kirkwood J.K., Simpson V.R., Robinson R.A. (2014). Epidemiological evidence that garden birds are a source of human salmonellosis in England and Wales. PLoS ONE.

[16] Smith O., Snyder W.E., Owen J.P. (2020). Are we overestimating risk of enteric pathogen spillover from wild birds to humans?. Biol. Rev. Camb. Philos. Soc..

[17] Ferens W.A., Hovde C.J. (2011). Escherichia coli O157:H7: Animal reservoir and sources of human infection. Foodborne Pathog. Dis..

[18] Stein R.A., Katz D.E. (2017). *Escherichia coli*, cattle and the propagation of disease. FEMS Microbiol. Lett..

[19] Tuner K., Moura C.N., Hajmeer M., Barnes A., Needham M. (2019). Overview of leafy greens—Related foodsafety incidents with a California Link: 1996 to 2016. J. Food Prot..

[20] Renu M., Yadav A.S., Tripathi V., Singh R.P. (2011). Seasonal effect on the shedding pattern of *Salmonella*, Escherichia coli and *Campylobacter* in Poultry. J. Vet. Public Health.

[21] Greig J., Rajić A., Young I., Mascarenhas M., Waddell L., LeJeune J. (2014). A scoping review of the role of wildlife in the transmission of bacterial pathogens and antimicrobial resistance to the food chain. Zoonoses Public Health.

[22] Vogt N.A., Pearl D.L., Taboada E.N., Mutschall S.K., Janecko N., Reid-Smith R., Bloomfield B.B., Jardine C.M. (2018). Epidemiology of *Campylobacter, Salmonella* and Antimicrobial Resistant *Escherichia coli* in Free-Living Canada Geese (Branta Canadensis) from Three Sources in Southern Ontario. Zoonoses Public Health.

[23] Gordus A.G., Mandrell R., Atwill E.R. (2011). Wildlife Survey for E. coli O157:H7 and Salmonella in the Central coastal counties of California. Center for Produce Safety Final Project Report 2009.

[24] Gorski L., Parker C.T., Liang A., Cooley M.B., Jay-Russell M.T., Gordus A.G., Atwill E.R., Mandrell R.E. (2011). Prevalence, Distribution, and Diversity of *Salmonella enterica* in a Major Produce Region of California. Appl. Environ. Microbiol..

[25] Steele M., Odumeru J. (2004). Irrigation water as source of foodborne pathogens on fruit and vegetables. J. Food Prot..

[26] Fonseca J.M., Fallon S.D., Sanchez C.A., Nolte K.D. (2011). *Escherichia coli* survival in lettuce fields following its introduction through different irrigation systems. J. Appl. Microbiol..

[27] Bédard E., Laferrière C., Déziel E., Prévost M. (2018). Impact of stagnation and sampling volume on water microbial quality monitoring in large buildings. PLoS ONE.

[28] Benton T.G., Bryant D.M., Cole L., Crick H.Q.P. (2002). Linking agricultural practice to insect and bird population: A historical study over three decades. J. Appl. Ecol..

[29] Black E.P., Hinrichs G.J., Barcay S.J., Gardner D.B. (2018). Fruit flies as potential vectors of foodborne illness. J. Food Prot..

[30] Liu H., Whitehouse C.A., Li B. (2018). Presence and Persistence of Salmonella in Water: The Impact on Microbial Quality of Water and Food Safety. Front. Public Health.

[31] Sassi H.P., van Ogtrop F., Morrison C.M., Zhou K., Duan J.G., Gerba C.P. (2020). Sediment re-suspension as a potential mechanism for viral and bacterial contaminants. J. Environ. Sci. Health Part A Toxic/Hazard. Subst. Environ. Eng..

[32] Gaertner J.P., Mendoza J.A., Forstne M.R.J., Hahn D. (2011). Recovery of *Salmonella* from biofilms in a headwater spring ecosystem. J. Water Health.

[33] Sha Q., Gunathilake A., Forstner M.R., Hahn D. (2011). Temporal analyses of the distribution and diversity of *Salmonella* in natural biofilms. Syst. Appl. Microbiol..

[34] Liu C., Hofstra N., Franz E. (2013). Impacts of climate change on the microbial safety of pre-harvest leafy green vegetables as indicated by *Escherichia coli* O157 and *Salmonella* spp.. Int. J. Food Microbiol..

[35] Satterfield D.A., Marra P.P., Sillet T.S., Altizer S. (2018). Responses of migratory species and their pathogens to supplemental feeding. Philosophical transactions of the Royal Society of London. Biol. Sci..

[36] Navarro-Gonzalez N., Wright S., Aminabadi P., Gwinn A., Suslow T.V., Jay-Russell M.T. (2020). Carriage and Subtypes of Foodborne Pathogens Identified in Wild Birds Residing near Agricultura Lands in California: A repeated cross-sectional Study. Appl. Environ. Microbiol..

[37] Verbrugghe E., Boyen F., Gaastra W., Bekhuis L., Leyman B., Van Parys A., Haesebrouck F., Pasamans F. (2012). The complex interplay between stress and bacterial infection in animals. Vet. Microbiol..

[38] Mootian G., Wu W.H., Matthews K.R. (2009). Transfer of *Escherichia coli* O157:H7 from soil, water, and manure contaminated with low numbers of the pathogen to lettuce plants. J. Food Prot..

[39] Zhu L., Juneja V.K., Fonseca J.M., Ravishankar S. (2015). Survival of *Escherichia coli* O157:H7 on lettuce harvested from fields irrigated by different irrigation systems and stored under different conditions. J. Agric. Life Sci..

[40] Fonseca J.M. (2006). Postharvest quality and microbial population of head lettuce as affected by moisture at harvest. J. Food Sci..

[41] Jeamsripong S., Chase J.A., Jay-Russell M.T., Buchanan R.L., Atwill E.R. (2019). Experimental In-Field Transfer and Survival of *Escherichia coli* from Animal Feces to Romaine Lettuce in Salinas Valley, California. Microorganisms.

